# Methods to Assess Proliferation of Stimulated Human Lymphocytes In Vitro: A Narrative Review

**DOI:** 10.3390/cells12030386

**Published:** 2023-01-20

**Authors:** Nirosha Ganesan, Steven Ronsmans, Peter Hoet

**Affiliations:** 1 Laboratory of Toxicology, Unit of Environment & Health, Department of Public Health and Primary Care, KU Leuven, 3000 Leuven, Belgium; 2 Laboratory of Respiratory Diseases and Thoracic Surgery (BREATHE), KU Leuven, 3000 Leuven, Belgium; 3 Clinic for Occupational and Environmental Medicine, Department of Respiratory Diseases, University Hospitals Leuven, 3000 Leuven, Belgium

**Keywords:** lymphocyte proliferation, LPT, flow cytometry, carboxyfluorescein succinimidyl ester, in vitro

## Abstract

The ability to monitor lymphocyte responses is critical for developing our understanding of the immune response in humans. In the current clinical setting, relying on the metabolic incorporation of [^3^H] thymidine into cellular DNA via a lymphocyte proliferation test (LPT) is the only method that is routinely performed to determine cell proliferation. However, techniques that measure DNA synthesis with a radioactive material such as [^3^H] thymidine are intrinsically more sensitive to the different stages of the cell cycle, which could lead to over-analyses and the subsequent inaccurate interpretation of the information provided. With cell proliferation assays, the output should preferably provide a direct and accurate measurement of the number of actively dividing cells, regardless of the stimuli properties or length of exposure. In fact, an ideal technique should have the capacity to measure lymphocyte responses on both a quantitative level, i.e., cumulative magnitude of lymphoproliferative response, and a qualitative level, i.e., phenotypical and functional characterization of stimulated immune cells. There are many LPT alternatives currently available to measure various aspects of cell proliferation. Of the nine techniques discussed, we noted that the majority of these LPT alternatives measure lymphocyte proliferation using flow cytometry. Across some of these alternatives, the covalent labelling of cells with a high fluorescence intensity and low variance with minimal cell toxicity while maximizing the number of detectable cell divisions or magnitude of proliferation was achieved. Herein, we review the performance of these different LPT alternatives and address their compatibility with the [^3^H] thymidine LPT so as to identify the “best” alternative to the [^3^H] thymidine LPT.

## 1. Introduction

Monitoring antigen-specific lymphocyte migration and proliferation is an essential way to acquire an understanding of the underlying mechanisms involved in human immune-mediated diseases [1] such as granulomatous diseases. As mediators of adaptive immunity, lymphocytes have the chemotactic ability to migrate throughout the body and position themselves within specific areas of lymphoid organs according to their states of differentiation [2], following antigen recognition. The rapid proliferation of antigen-specific T cell and B cell subpopulations ensues when coming into contact with an antigen, resulting in a consequential change in the further migration, activation, and proliferation patterns of responding cells. To analyze the complex series of events that follow suit, precise procedures and methods are necessary to not only track the proliferation history of the lymphocytes studied [2], but also simultaneously identify which subpopulations are directly involved.

Due to their convenient accessibility, antigen-specific responses are preferentially evaluated in vitro with the use of peripheral blood mononuclear cells (PBMCs), for which lymphocytes (70–90%) make up the bulk of the population [3]. Traditional methods of determining sensitization in affected individuals include prick skin testing, intradermal skin testing, IgE radioallergosorbent tests, and patch testing [4]. However, each of these methods has their own drawbacks, such as unexpected irritant reactions and sensitization by patch testing [5]. On the other hand, the blood lymphocyte proliferation test (LPT) based on [^3^H] thymidine incorporation is a non-invasive technique that measures cell-mediated T cell responses to specific antigens. It serves as a viable tool in the clinical evaluation and medical surveillance of exposed or at-risk workers from industries such as mining, construction, and iron foundries, as well as patients with T cell dysfunction, i.e., T cell immunodeficiency. The use of an LPT as a clinical screening method is therefore recognized to be effective for both the early identification of disease risk and the secondary prevention of diseases [4].

However, the use of a radioactive material such as [^3^H] thymidine can be potentially biohazardous and, moreover, suffers from several drawbacks [2]. Firstly, as an endpoint assay, it is impossible to follow the migration or proliferation behavior of individual cells, and no information on the extent of the cellular divisions undergone or the division history of proliferated cells can be obtained [2]. Although [^3^H] is a less damaging isotope as compared to gamma-emitting isotopes such as [^51^Cr] and [^125^I], it is still a laborious effort to detect the presence of these isotopes due to their susceptibility to quenching and possible low emission energies [2]. Additionally, it is also difficult to determine and distinguish if the radioactivity recovered at the end of the exposure period is associated with responding and proliferating cells alone or if it also reflects the incorporation of [^3^H] thymidine into the DNA strands of bystander cells [2]. This would be a major concern in studies focused on the cytotoxic influence of antigens on lymphocyte proliferation. 

Strictly speaking, the labelling of lymphocyte populations should ideally be easily performed with labels that are non-toxic, non-transferrable to other cells, able to remain stable for several days or even weeks, and able to remain easily detectable. In an attempt to overcome the setbacks associated with the use of the [^3^H] thymidine LPT and identify ideal alternatives, the use of fluorochromes to track lymphocyte proliferation was proposed. 

With the use of fluorescence microscopy, fluorescent cells in cell suspensions have been enumerated and quantified [6]. At the same time, developments in the flow cytometry field have opened up new possibilities for both the phenotypical and functional characterization of lymphocyte subpopulations and their antigen-specific responses [7]. As a result, a considerable range of fluorescent dyes, antigens, and markers were made available for lymphocyte tracking, proliferation, and migration studies [2], and these very quickly replaced the tedious process of quantifying fluorescent cells with microscopy. A majority of these flow-cytometric-reliant dyes and markers were therefore tested for their robustness and efficacy as alternatives to the [^3^H] thymidine LPT, i.e., LPT alternatives.

In this review, various LPT alternatives that incorporate the use of either fluorescent dyes or immunophenotyping markers were elaborated on and grouped according to their staining mechanism. Comparative studies evaluating the correlation between the [^3^H] thymidine LPT and the respective LPT alternatives were additionally selected to facilitate a discussion on whether, and if so, to what extent, a better LPT alternative to [^3^H] thymidine incorporation exists and can contribute to the field.

## 2. Results and Discussion

### 2.1. Applications of LPT

The proliferative capacity of immune cells or lymphocytes is most frequently determined by the incorporation of a radiolabeled DNA precursor into newly generated DNA, known as tritiated thymidine (^3^H-TdR; [^3^H] thymidine) [8]. [^3^H] thymidine is used for exclusively marking replicating DNA, since it is a DNA precursor that is also at the same time not involved in RNA synthesis [9].

**Figure 1:**
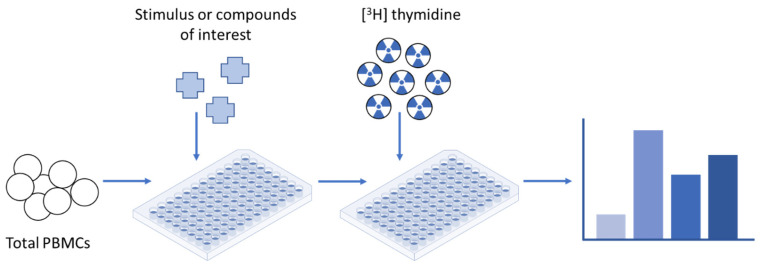
Lymphocyte proliferation assay—[^3^H] thymidine incorporation. The classic thymidine incorporation assay or lymphocyte proliferation test (LPT) relies on the use of a radioactive nucleoside, [^3^H] thymidine, that is incorporated into newly dividing strands of DNA during mitotic cell division. Radioactivity in DNA is then recovered from peripheral blood mononuclear cells (PBMCs) and measured to determine the extent of cell division/proliferation that has occurred in response to any antigen of interest.

To be precise, lymphocytes, or more specifically, peripheral blood mononuclear cells (PBMCs), are stimulated and maintained in culture for several days with antigens at varying concentrations. After a specified time, typically 4–6 days after initiation, [^3^H] thymidine is added to the cell culture. Proliferation is then assessed by the degree of the cellular uptake of [^3^H] thymidine (Figure 1), termed as counts per minute (cpm), and is indicative of cells undergoing DNA replication in the S-phase of the cell cycle. The results are expressed as a stimulation index (SI)—a ratio of radiolabeled thymidine uptake in the stimulated and antigen-exposed cells compared to the uptake in the unstimulated cells [10].

A positive test is generally identified by two or more SIs across a range of concentrations that exceed a specific threshold of abnormal values, which is known as the SI threshold [4]. The SI threshold typically either exceeds a specific cut-off, i.e., SI ≥ 1.5–3.0 in some laboratories, or relies on the statistical determination of a mean peak SI among unexposed, non-sensitized subjects [11]. 

Hence, a test may be interpreted as normal if none of the concentrations elicit an SI value above the cut-off. Concurrently, when the SI of only one of the antigen concentrations is elevated above the cut-off, the test is then regarded as “borderline” (BL) [4], which then warrants a repeat test to be performed.

However, in current clinical practice, the [^3^H] thymidine LPT is only routinely used either to detect patients with T cell immunodeficiency or as a measure of immunologic screening for sensitization to beryllium. 

#### 2.1.1. T Cell Immunodeficiency

T cell immunodeficiency can occur as part of a group of primary disorders, which is known as primary T cell immunodeficiency, or develop secondary to chronic infections such as the human immunodeficiency virus (HIV), the Epstein–Barr virus (EBV), malaria, or illnesses such as diabetes or liver/renal failure [12]. Primary T cell immunodeficiencies are typically the result of a defective immune system from infancy and early childhood [12], while secondary T cell immunodeficiency is a consequence of different causes such as drugs, infections, malnutrition, or systemic diseases [12]. 

Patients with mitogen-induced lymphocyte proliferation that is <10% of normal healthy controls are defined as having a profound T cell deficiency. These patients, specifically infants, are then regarded as candidates for hematopoietic stem cell transplantation (HSCT) to reconstruct immunity and prevent them from life-threatening opportunistic infections [13]. Lymphocyte proliferation and neonatal screening for the T cell receptor excision circle (TREC) in the Guthrie card are the two main methods relied upon to identify patients with profound T cell defects [13]. At times, the exclusion of patients with a profound T cell deficiency can be achieved based on an acceptable level of lymphocyte proliferation, despite a low TREC value observed in their respective Guthrie cards [13]. In that aspect, the LPT is thus valued as a sensitive and reliable method to detect a reduced lymphocyte proliferation in patients with T cell immunodeficiency, and more so in patients with unknown genetic defects that may also gradually develop a T cell deficiency as they age [13]. 

#### 2.1.2. Metal-Specific LPTs

Metals are known to cause a number of different pathological conditions, and the inhalation of metal dust can cause a variety of lung diseases, such as parenchymal lung fibrosis and granulomatous lung disorders. The increased industrial use of beryllium and the consequent occupational beryllium exposure has resulted in a rise in patients suffering from beryllium sensitivity (BeS), berylliosis, or chronic beryllium disease (CBD)—a lung granulomatous disease caused by a cell-mediated immune response to beryllium (Be). CBD manifests as a granulomatous lung disease, and is generally diagnosed based on the demonstration of a sensitization to Be and the presence of non-caseating granulomas on a lung biopsy [10].

Be is the second lightest metal with unique physical and chemical properties that allow for Be metal, Be-containing alloys, and beryllium oxide (BeO) to be utilized in high-technology industries such as aerospace, telecommunications, ceramics, electronics, and defense [14]. Be exposure primarily occurs through inhalation, mostly in workers of high-risk groups such as Be alloy machinists and construction workers, in spite of the stringent efforts made to conduct periodic medical surveillance in workers [15]. Although the majority of Be-exposed workers develop no pressing health problems, Be is still regarded as a carcinogen of the utmost importance [15]. 

Workplace screening for BeS using the blood beryllium lymphocyte proliferation test (BeLPT) identifies workers with clinically significant and/or symptomatic CBD as well as workers with early evidence of granulomatous lung disease in the absence of symptoms at the time of the initial clinical evaluation—beryllium-sensitized (Be-sensitized) [16]. Since the 1980s, BeLPT has been utilized as a means of immunologic screening for CBD, and has identified CBD cases in workers prior to the presentation of clear symptoms that are recognized as being related to beryllium exposure [16]. The BeLPT has since been regarded as a gold standard and incorporated as part of the USA beryllium workforce medical surveillance [17] due to its involvement in clinically differentiating CBD from other lung granulomatous diseases, as well as its ability to allow for the identification of individuals at a significant risk of disease progression [8] or BeS. Be-sensitized and CBD patients can then be further confirmed with an evaluation via a bronchoscopy with bronchoalveolar lavage (BAL) and a transbronchial biopsy.

Studies or case reports have since shown that occupationally-acquired sensitization from metals has presented opportunities for the utilization of an LPT in better comprehending the underlying pathophysiologic mechanisms and in providing more accurate diagnosis based on assessment of occupational sensitization [4]. These case reports have also demonstrated an increased lymphocyte proliferation to titanium [18], aluminum [19,20], chromium, and nickel [21] in affected individuals.

However, the incidence rate of complications in the lung or pulmonary diseases in workers exposed to metals apart from beryllium is still less frequently reported. This is mostly due to the lack of a similarly validated test such as the BeLPT [22], and also because no other metal-specific LPT has thus far been introduced as a routine measure in medical surveillance programs to determine the sensitization in exposed workers or individuals. The only exception to this is seen in the case of the nickel LPT (NiLPT)—a recent advance in the use of the LPT—to identify nickel hypersensitivity based on the dysfunction of surgical prostheses in patients undergoing initial joint replacement surgery at National Jewish Health [23]. This field represents a new area of toxicologic assessments of metal allergies with potential occupational surveillance applications, and it explores interactions with metal ions such as nickel, cobalt, chromium, and even titanium/aluminum alloys found in orthopedic implants that release 0.5–1% nickel as breakdown products into tissues and circulation [21,24].

Nonetheless, there is still a lot of potential in the use of other metal-specific LPTs in this field, although there is still a skeptical outlook on whether such LPTs can be equally specific and sensitive as the BeLPT. 

### 2.2. LPT Alternatives

The LPT is a highly specific and sensitive tool for assessing the sensitization to particular occupational agents, although it does come with several limitations. The limitations include the length of time required to run the assay, exposure to radiation for workers performing the test, and a lack of information on the viability of cells in the cell culture or the type of responding lymphocyte subsets present [25]. Moreover, quantifying incorporated [^3^H] thymidine in dividing cells does not give any intrinsic information about the extent of the divisions observed in individual cells [26]. Additionally, the use of the LPT has been heavily scrutinized due to reports of inter- and intra-variability in results, observed between laboratories performing the test [27]. 

Improvements to the LPT that allow for the elimination of dead cells from the analysis could play a vital role in reducing inter-laboratory variability. In addition, the ability to measure lymphocyte proliferation within specific lymphocyte subsets would help to characterize the immune response per individual. If a strong association between the disease of interest and a specific subset of lymphocytes can be drawn, then the results from immunolabeling simultaneously with proliferation markers may enable a prediction of disease development and a diagnosis of the disease [8]. 

#### 2.2.1. Proliferation Assays

As such, over the last decades, several alternatives have been proposed to address the abovementioned shortcomings of the LPT, and they have been assessed across laboratories where radioactive facilities are limited or absent. These alternatives include the memory lymphocyte immunostimulation assay (MELISA^®^) [28] and flow-cytometry-based assays such as the bromodeoxyuridine/5-bromo-2′-deoxyuridine (BrdU) assay [8], the ethynyldeoxyuridine/5-ethynyl-2′-deoxyuridine (EdU) assay [29], the carboxyfluorescein succinimidyl ester (CFSE) assay [13,30,31,32,33,34,35,36,37,38,39], the flow-cytometric assay for specific cell-mediated immune response in activated whole blood (FASCIA) [40], PKH26 dye [2], and violet proliferation dye 450 (VPD-450) [1]. These techniques have been proven to identify proliferative responses without the need for radioactive agents, at single or multiple time points, and they have the ability to be run on commercial clinical flow cytometers, in turn improving the accessibility to more researchers to evaluate the lymphoproliferative capacity of cells [4]. 

Despite the advent of high-affinity T and B lymphocyte markers, the above-mentioned techniques, especially flow-cytometry-based assays such as the BrdU assay, the CFSE assay, and the use of VPD-450, are still preferentially used to evaluate T cell proliferation only. As such, there are only limited studies that clearly make the distinguishment between T lymphocytes and overall lymphocytes, i.e., T and B lymphocytes combined. Regardless, activation markers—clusters of differentiation—69 (CD69^+^), CD25^+^ [38,39], human leukocyte antigen–DR isotype (HLA-DR^+^), proliferation marker Ki67^+^ [13], and colorimetric assay-3-(4,5-dimethyl thiazol-2-yl)-2,5-indiphenyl tetrazolium bromide (MTT, [41]) have also been used to a lesser extent, in conjunction with some of the flow-cytometry-based assays and the LPT or even independently, to determine lymphocyte proliferation. The correlations between the LPT and these alternatives have also been evaluated extensively (Table 1), allowing researchers in the field today to easily rely on one or the other technique.

##### Memory Lymphocyte Immunostimulation Assay (MELISA^®^)

A modification of the LPT for detecting metal sensitivity was devised by Stejskal and colleagues in 1994, known as the memory lymphocyte immunostimulation assay (MELISA^®^, [47]). Such an alternative was developed at the request of the Swedish pharmaceutical company Astra, as they required a test for the diagnosis of occupational drug allergies in their drug-producing workers who were presumably exposed to dust containing Be.

As a result, the methodology of the LPT was optimized to develop MELISA^®^ with an improved specificity and sensitivity, which was achieved by (1) utilizing a higher number of lymphocytes per test, (2) selecting concentrations that are non-cytotoxic and non-mitogenic, (3) partially depleting monocytes from the lymphocyte population, and (4) confirming the [^3^H] thymidine-based radiological result with a morphological examination [48]. Since its advent, several countries across the globe have been licensed to perform MELISA^®^ testing, and several studies have been published to demonstrate its clinical utility [24,28,42,49]. By doing so, the reproducibility and reliability of this test was enhanced, and it is thus recognized as a clinically validated blood test for detecting hypersensitivity to multiple metals. 

The testing panels offered by MELISA^®^ diagnostics include individual testing of metals such as aluminum (Al), beryllium (Be), cadmium (Cd), chromium (Cr), cobalt (Co), copper (Cu), gold (Au), lead (Pb), manganese (Mn), nickel (Ni), palladium (Pd), platinum (Pt), silver (Ag), tin (Sn), titanium (Ti), tungsten (W), vanadium (V); testing for antigens such as *Candida albicans* (*C*. *albicans*); and testing for silicon dioxide (SiO_2_). 

Based on the MELISA^®^ guidelines, an SI < 2 was considered negative, an SI between 2 and 3 was interpreted as “possible sensitization” [48] or a weak positive test result, and an SI > 3 was seen as a positive test result. In some MELISA^®^ studies, an SI cut-off of SI ≥ 5 [50] is also applied instead of SI ≥ 3, resulting in an improved reproducibility rate. By relying on these guidelines, several studies also chose to evaluate the sensitivity of beryllium-specific MELISA^®^ in comparison to BeLPT [28,42]. Beijer et al. observed a perfect correlation (*r* = 1.0, *p* < 0.01) between the BeLPT and the beryllium SI of MELISA^®^ across four patients [42], while Fireman and colleagues observed concordance between a positive BeLPT and a positive test result with beryllium-specific MELISA^®^ in a single patient [28]. 

#### 2.2.2. Flow-Cytometry-Based Proliferation Assays

There are intrinsic advantages to replacing LPT—a bulk measurement assay that relies on the incorporation of radiochemical [^3^H] thymidine—with flow-cytometry-based assays [8]. The advantages include high-throughput single cell measurements that use fluorescent rather than radioactive reagents, the ability to evaluate cytotoxic effects of stimulants and derive information about changes in cell viability over time, and measuring the extent of the proliferation observed in specific lymphocyte subsets of interest [8].

Additionally, fixed samples can be saved for longer periods, i.e., 2–14 days before the flow cytometry analysis, the data acquired can be stored for reanalysis, and the reagent and labor costs are similar to, if not less than, the LPT. Unlike with the LPT, which requires the transferring of cells onto glass fiber filters for the purpose of cell harvesting and counting, samples labelled with fluorescent dyes or tags can be analyzed in a straightforward manner on most commercial clinical flow cytometers without the need for cell transfer [8].

##### Nucleotide-Based: Bromodeoxyuridine/5-Bromo-2′-deoxyuridine (BrdU) Assay

A halogenated synthetic nucleoside analogue of [^3^H] thymidine, bromodeoxyuridine/5-bromo-2′-deoxyuridine (BrdU), has also been used to quantify cell proliferation based on its incorporation into DNA during replication within the S-phase of the cell cycle [51]. The quantification of the incorporated BrdU has been considered the most direct measure of cell proliferation [51] and is also regarded as a valuable alternative technique for determining DNA replication by addressing the limitations of the LPT. BrdU incorporated into DNA is recognized by specific polyclonal or monoclonal antibodies produced against bromouridine or iododeoxyuridine. This ability to combine BrdU labelling with the detection of cell-type-specific markers has been equally regarded as a gold standard for studying cell division and differentiation in multicellular organisms [29].

However, similar to [^3^H] thymidine, BrdU is equally toxic, and its incorporation into DNA has unpredictable effects on cellular function and behavior [52]. The detection of BrdU necessitates DNA denaturation, which can disrupt the DNA integrity in the process [51] and inhibit cell cycle progression as well as limit the use of concurrent molecular assays [29]. Since the incubation of cells with BrdU is so short-lived, i.e., less than 24 h, only cells that have progressed through the S-phase would be detected [43]. Moreover, identifying the specific contribution of certain lymphocyte subsets to the overall proliferation signal is further limited [45]. 

Regardless, a good association based on the overall correlation between the BrdU assay and [^3^H] thymidine incorporation (*r* = 0.82–0.92) when stimulated with anti-CD3/CD28 monoclonal antibodies (mAbs) was observed in HIV-1-infected individuals and controls [45]. Concurrently, Farris and colleagues, having recognized the many limitations of solely relying on BrdU for the detection of proliferation, developed an improvised, yet simple, flow cytometric assay alternative, termed immuno-BeLPT, for the detection of beryllium [8]. Immuno-BeLPT encompasses a combination of known techniques, such as the BrdU assay to detect cell proliferation, surface immunolabelling, and 4′6′-diimidazolin-2-phenylindole (DAPI) or propidium iodide (PI) stains, to determine the DNA content [8]. A good agreement between the BrdU assay and staining with either DAPI or PI was observed in CBD patients, and based on these findings, they have also proposed the use of immuno-BeLPT as a follow-up test to the conventional BeLPT [8]. 

##### Nucleotide-Based: Ethynyldeoxyuridine/5-Ethynyl-2′-deoxyuridine (EdU) Assay

Ethynyldeoxyuridine/5-ethynyl-2′-deoxyuridine (EdU) is a thymidine analogue where the methyl group in thymidine is replaced by a terminal alkyne group [53]. The incorporated EdU is detected by a simple copper [Cu(I)]-catalyzed covalent reaction between the alkyne group of EdU and a fluorescently tagged azide [54], allowing for the rapid and efficient penetration of tissue sections and organs [55]. This assay requires only the standard detergent-based permeabilization of the cell and nuclear membranes, and as a result of the small size of the fluorescent azide probe, the EdU units within double-stranded DNA are easily accessible [51]. Therefore, the use of EdU does not involve the denaturation of DNA, hence overcoming the major drawback associated with the use of thymidine analogues [29]. 

As opposed to the BrdU assay, the EdU assay is fully compatible with nuclear counterstains and can be multiplexed with the antibody-based detection of other antigens of interest or other markers of DNA and cell cycle proliferation and progression. Furthermore, the fluorescent labelling of EdU is safe, as it does not interfere with biochemical reactions occurring in live cells [29], and the entirety of the procedure can be completed in less than an hour, thus allowing it to be both fast and effective [51]. EdU has thus far been utilized and developed into kits for flow cytometry and imaging applications such as immunohistochemistry (IHC), and has been adapted for colorimetric detection in an IHC assay. 

Although the incorporation of EdU into DNA does not interfere with the biochemical reactions in the cell per say, the cytotoxic effect of the catalyst—Cu(I) ions—in the EdU assay is assumed to be detrimental and to induce a heightened toxicity as opposed to BrdU and other assays. EdU is also believed to work within a limited concentration range, such that decreasing its dose would inevitably reduce the number of labelled cells [55]. Despite these limitations and the availability of commercial kits, it is necessary to note that the use of EdU as an alternative to either [^3^H] thymidine or BrdU incorporation has thus far only been evaluated in in vivo or in vitro studies that are unrelated to the evaluation of lymphocyte proliferation in humans [29,56,57].

##### Dye-Based: Carboxyfluorescein Succinimidyl Ester (CFSE) Assay

**Figure 2:**
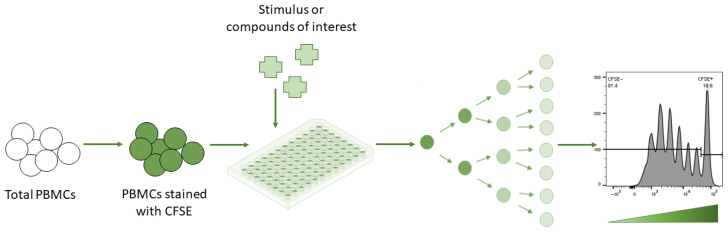
Lymphocyte proliferation assay—CFSE assay. Peripheral blood mononuclear cells (PBMCs) are labelled with a fluorescent cytoplasmic proliferation dye known as carboxyfluorescein succinimidyl ester (CFSE). Since the dye eventually dilutes out per generation of daughter cells upon cell division, each round of division can be tracked individually over time. This flow-cytometry-based analysis further allows for the simultaneous quantification and characterization of various individual cell types alongside proliferation determination.

Carboxyfluorescein succinimidyl ester (CFSE) is an effective dye-based means to monitor lymphocyte proliferation [58]. CFSE has the capacity to covalently bind long-lived intracellular molecules with the fluorescent dye carboxyfluorescein. Carboxyfluorescein-tagged molecules are measured based on their cell fluorescence via flow cytometry (Figure 2), and therefore, the progressive halving of CFSE fluorescence intensity per generation of daughter cells can be assessed [26]. The ability of CFSE to initially label lymphocytes with a high fluorescence intensity, coupled with its low cytotoxic effect, has made CFSE an ideal alternative to measure cell divisions or lymphocyte proliferation [58]. Due to it being a fluorescein-based dye, it is hence also compatible with other fluorochromes, allowing it to be easily modified for multi-color flow cytometry [58].

CFSE was initially utilized as a dye for tracking cell division in vivo [58] and regarded as a useful choice for long-term migration studies. Its utility has since expanded and has been validated across several in vitro studies [26] due to its potential for tracking up to eight cell divisions [26] in cells that have been stimulated by mitogens or antigens [59]. With the combination of monoclonal antibodies and CFSE dye, it is feasible to identify the phenotype and determine the precursor frequency of proliferating cells [59]. Due to the ease of modifying study designs to be tailor-made with the use of multiparameter flow cytometers, several studies have evaluated the efficacy of the CFSE assay as well as other flow-cytometric-based assays and have since advocated for the LPT to be replaced with the CFSE assay instead (Table 2).

The quantitative analysis of CFSE-labelled lymphocyte proliferation can be determined in several ways. Brinke and colleagues have provided a detailed breakdown on the different modes of analyzing proliferating cells, which more often than not relies on the number of distinct peaks of division. For instance, the extent of proliferation can be depicted as (1) the precursor frequency (PF)—the proportion of cells with a reactivity to the stimuli of choice within the starting population and (2) the proliferation index (PI)—the average number of cell divisions in responding cells [7]. Thus far, the experimental data have been evaluated through the quantitative parameters of the CFSE assay, which include the percentage of dividing cells [26,30,32,35,38,59], the percentage of non-proliferating cells [35], the percentage of dividing cells in relation to the total number of cells [26,33,38,59], and the number of cell divisions [7,30]. 

However, the easiest and most preferred way to determine the proliferative capacity of CFSE-labelled cells is by comparing the percentages of cells showing dye dilution, or a lower median fluorescence intensity (MFI), in stimulated and unstimulated conditions. This proportion of cells that have divided is also known as the percentage divided (% divided) or CFSE^dim^ [33]. 

As an equivalent to the LPT-derived SI, the CFSE assay-based SI is therefore a ratio of the % divided in stimulated conditions over the % divided in unstimulated conditions:

(1) $$\frac{stimulated\;divided}{stimulated\;total}\left(\%\right)/\frac{unstimulated\;divided}{unstimulated\;total}\left(\%\right)=\mathrm{CFSE}\;\mathrm{derived}\;\mathrm{SI}$$

Due to the advent of the CFSE assay as a promising LPT alternative, several studies have focused on comparing these two methods by stimulating PBMCs with phytohemagglutinin (PHA [32,38]), pertussis vaccinal antigens (PT) [30], tetanus toxoid (TT, [37]), *C. albicans* [39], and even beryllium [33]. Across all of these studies, a consistently strong and significant correlation was observed for the cpm of the LPT when compared with the % divided (*r* = 0.69–0.78, *p* < 0.05; [30,32]), and weighted % divided (*r*^2^ = 0.99, [39]). A good and significant agreement between both methods (0.627–0.805) was also noted in Be-stimulated PBMCs from controls as well as CBD and Be-sensitive patients [33]. The CFSE assay also yielded stronger proliferative responses than the LPT when stimulated with TT [37], and it also allowed for a clearer discrimination between responders and non-responders [38] in PHA-stimulated PBMCs.

##### Dye-Based: Flow-Cytometric Assay for Specific Cell-Mediated Immune Response in Activated Whole Blood (FASCIA)

In 1996, Gaines and colleagues developed a new method for measuring lymphocyte proliferation using whole blood and the flow-cytometric identification of replicating lymphoblasts [64], known as the flow-cytometric assay for specific cell-mediated immune response in activated whole blood (FASCIA). FASCIA measures the blast formation of proliferating lymphocytes [40], and the cut-off for a FASCIA-positive response is expressed as the number of cells that have proliferated in response to a stimulus per µL of whole blood (cells/µL). Since the lymphocyte size increases during cell division, and this can be easily detected with an increase in forward scatter via flow cytometry, no specific dyes are utilized in this assay [40]. The read-out of blast-transformed cells can be additionally combined with lymphocyte markers, enabling the enumeration of responding/proliferating lymphocyte subpopulations [40] and making it an easily versatile and comparable technique even with other flow-cytometry-based assays. A major advantage of working with whole blood is that the time-consuming and sometimes complex step of separating PBMCs from peripheral whole blood is omitted. 

The additional benefits of FASCIA as opposed to a PBMC-dependent analysis include the following: (1) a smaller volume of blood is required; (2) it is easier to perform since fewer manipulations are involved; (3) it is less time-consuming and requires fewer resources, translating to reduced overall costs; and most importantly, (4) the immune cells in whole blood are maintained in an environment resembling that found in vivo. 

As a result, FASCIA has already been introduced and implemented in some laboratories, including the laboratory of clinical immunology at the Karolinska University Hospital, as a diagnostic test for various types of immunodeficiencies following validation against 100 healthy controls [40]. The detailed protocol for the FASCIA procedure is available at the COST action BM0907 ENTIRE website (Action BM0907—COST). 

With mitogens such as PHA (*r* = −0.36, *p* = 0.38) and PPD (*r* = 0.82, *p* = 0.04), and antigens such as TT (*r* = 0.71, *p* = 0.08) and cytomegalovirus (CMV; *r* = 0.63, *p* = 0.12), induced responses with the use of FASCIA were also correlated with those of the LPT [64]. At the same time, the proliferative responses appeared to be more specific with antigens such as CMV, and higher SI values were generated with FASCIA when compared with the LPT [64]. 

##### Dye-Based: Paul Karl Horan (PKH) Dyes

In the late 1980s, Paul Karl Horan (PKH) discovered and patented several membrane-inserting fluorescent dyes—PKH2, PKH26, and PKH67—in an attempt to address several pitfalls of existing techniques, as well as to efficiently monitor lymphocyte trafficking and proliferation [2]. These dyes, being nonspecific, are able to label a wide variety of cell types, and they are non-cytotoxic and cause no adverse effects to the biological or proliferative activity of cells. Just as with CFSE, PKH dyes halve the cellular fluorescence per generation of daughter cells, allowing the fluorescence intensity to be readily captured by flow cytometry [65,66]. Moreover, the additional advantages of PKH dyes when compared to CFSE include showing little to no toxicity [67], an ability to monitor the proliferation of haemopoietic stem cells [68], the in situ labelling of spleen cells [69] or peripheral blood neutrophils [70], and their ability to track lymphocyte migration in vivo from weeks to even months [71,72]. 

However, the exact structure of PKH dyes is unclear, and when compared with CFSE, PKH26 for instance stains lymphocyte populations less homogenously than CFSE, resulting in less discrete peaks of proliferating or dividing PKH-labelled cells than CFSE-labelled cells [2,61]. Despite the limited studies that have compared the use of PKH dyes with other techniques, a good correlation between the proportion of cells in the S-phase denoted by BrdU incorporation and the PKH67 fluorescence decrease was noted in a study that aimed to compare cell proliferation in acute myeloid leukemia patients [73].

##### Dye-Based: Violet Proliferation Dye 450 (VPD-450)

Violet proliferation dye 450 (VPD-450), also rebranded as Cell Trace^TM^ Violet (CTV) [74], is a violet laser-excitable dye that can be used for the flow-cytometric monitoring of the cell divisions of single cells. Similar to CFSE, the nonfluorescent VPD-450 dye easily diffuses across cell membranes and is cleaved by intracellular esterases [75], resulting in a highly fluorescent compound that is well retained in viable cells following the covalent binding to intracellular amines [75,76]. As viable cells divide, the VPD-450 fluorescence is distributed uniformly and halved per generation of daughter cells [75,76]. 

A major advantage of relying on VPD-450 or CTV rather than CFSE is that this dye emits maximally at 450 nm with minimal spillover into the fluorescein isothiocyanate (FITC) channel. Therefore, the simultaneous detection of green fluorescent protein (GFP)-tagged cells or the use of other multi-color assays that require measurements in the FITC channel can be performed [77,78], since FITC-conjugated antibodies are more frequently manufactured. 

When selecting the optimal proliferation dye for their experimental setup, Brinke and colleagues validated and analyzed the effects on cells in a preliminary set of experiments, with the use of various concentrations of both CFSE and VPD-450 [7]. Increasing the concentrations of both CFSE and VPD-450 led to a higher fluorescence intensity observed, but unlike with CFSE, no change in cell viability were noted with VPD-450 concentrations < 5 µM. At lower concentrations of 1 and 2 µM, a better peak separation was observed with VPD-450 rather than CFSE, ultimately leading to the selection of VPD-450 in their study [7]. Therefore, the preferential selection of VPD-450 for CFSE is only observed when laboratories perform a selection procedure to determine which dyes are best suited based on their choice of reagents, media, and access to flow cytometers with two or more lasers. 

A comparison of the proliferation data from a VPD-450 dye-based assay and the LPT additionally showed a good correlation in response to allogenic antigens and pathogen-derived antigens such as *C*. *albicans*, TT, and the *Varicella zoster* virus (*V*. *zoster* virus) (*r* = 0.91, *p* < 0.0001, [7]). A comparable sensitivity in detecting reactive T cells of a low frequency to autologous antigens between both techniques was also noted [7].

#### 2.2.3. Flow-Cytometry-Based Immunophenotyping

Immunophenotyping is a technique used to profile immune cells by coupling antibodies of interest to known fluorescent compounds to measure the protein expression—both on the cell surface and intracellularly—of a specific population of cells. Moreover, this technique can be performed not only in peripheral blood, but also in tissues and even tumor samples. Labelled cells, such as CD3 or CD4 T cells, are then acquired via flow cytometry and their expression levels are compared to study specific immune-related diseases or immunodeficiency disorders. 

##### Activation Markers: CD69^+^, CD25^+^, and HLA-DR^+^

Both the activation and proliferation of immune-competent cells are recognized as equal contributors to a successful immune response against foreign antigens [79]. More often than not, the simultaneous measurement of activation markers—CD69, CD25, and HLA-DR—are frequently implemented alongside proliferation dye-based flow-cytometric analyses as a measure of lymphocyte activation and proliferation, respectively [38,39]. 

Within a stipulated exposure period, the responsiveness of T lymphocytes to mitogens and antigens can be analyzed by measuring early CD69^+^, middle CD25^+^ (also known as the interleukin-2 (IL-2) receptor [80]), and late HLA-DR^+^ T lymphocyte markers on CD3^+^ T cells [81]. Although the expression of these markers presumably indicates progression through the cell cycle stages, it is in fact more frequently associated with progression through time in stimulated cell cultures [38]. 

For instance, the early activation marker CD69^+^ is expressed immediately after T cell activation upon both antigen and mitogenic stimulation. Its frequency can remain upregulated for 4 h [81] before peaking at 24 h [38] prior to initiating the transcription of IL-2 and the tumor necrosis factor (TNF) [82,83]. However, studies have reported that the expression of CD69^+^ progressively declines per cell division [38], further substantiating that the expression of these activation markers is time-dependent. 

CD25^+^ is also upregulated between 12 and 24 h of antigenic stimulation, and the maximal levels of activation can even be observed several days later [38,81]. Cell proliferation and differentiation are typically initiated following the heightened expression of CD25^+^ [84]. As opposed to CD69^+^, however, the expression of CD25^+^ remained active throughout the culture period, and remained proportional to the antigen dosage [38]. Moreover, both antigenic and mitogenic stimuli induced the CD25^+^ expression on a larger number of lymphocytes when compared with CD69^+^ or HLA-DR^+^ expression [79]. Therefore, CD25^+^ is the most frequently used activation marker to detect possible low-frequency responses and to study immune responses in patients with a reduced number of T lymphocytes [79]. 

A less frequently used marker, but a good indicator of late or delayed activation, is the upregulation of HLA-DR^+^, which is usually observed between 48 and 60 h after antigenic stimulation. The expression of HLA-DR^+^ highlights progression towards adaptive immunity [81]. 

Caruso et al. showed that by modulating the stimulus dosage or by considering different time points, the expression of activation markers on T lymphocytes is still induced, while no change in proliferation—evaluated by [^3^H] thymidine or BrdU incorporation—was found when compared with unstimulated controls [79]. As a result, no correlation between the CD69^+^, CD25^+^, and HLA-DR^+^ expression and lymphocyte proliferation with mostly mitogens was noted [79]. On the other hand, Maino and colleagues observed that the CD69^+^ expression was comparable with the [^3^H] thymidine incorporation when PBMCs were stimulated with anti-CD2/CD2R mitogenic mAbs [85]. It is hence plausible that the extent of the activation and proliferation observed would rely on the nature of the antigenic or mitogenic stimuli.

Therefore, combining the variations in the proportions of T cell activation observed with different stimuli and their association with the extent of proliferation observed can provide additional insights into the events that prelude cell-mediated immune responses across more diseases. 

##### Proliferation Marker: Ki-67^+^

Ki-67 is a nuclear protein that plays a role in the regulation of cell division. The mAb marker Ki-67^+^ is expressed during all active phases of cell division (proliferating cells), the G1-, G2-, S-, and M-phases, and is absent in quiescent cells (resting cells), G0, and during DNA repair [86]. As a result, Ki-67 appears to be more sensitive as a marker of proliferation for the detection of rare T cell responses, and in turn, more accurately reflects the extent of in vitro antigen-induced proliferation [43] as opposed to LPT, BrdU, or EdU assays. The intracellular Ki-67^+^ expression has been used to measure specific T cell responses induced by vaccination [87,88] and chronic viral infections such as HIV infections [89,90], and is also extensively involved in tumor cell proliferation [91] and the prediction of cancer prognoses [92]. 

As a technique, the Ki-67 proliferation assay has proven to be time- and cost-effective, as it does not require any incubation or washing steps before or during culture, thereby eliminating the exposure of cells to possible toxic dye compounds in the process [43]. At the same time, its inability to account for the number of proliferation cycles that stimulated cells have undergone, unlike its dye dilution assay counterparts, limits its utility [2]. Regardless, researchers have since quantified the expression of Ki-67 as a proliferation index (PI, [92]) which is defined as the percentage of Ki-67^+^-positive cells present. The evaluation of the PI is routinely conducted during histopathological examinations, especially in cases [93] for which the PI is also regarded as a predictive biomarker, i.e., breast cancer [94] and gastric cancer [92]. As an alternative technique to the SI of the LPT, lymphocyte proliferation is denoted as the Ki-67^+^ PI in unstimulated wells subtracted from the Ki-67^+^ PI in stimulated wells [13]. 

However, in patients with a profound T cell deficiency, Lee et al. noted a non-significant correlation between the LPT, the CFSE assay, and Ki-67 staining [13], despite the fact that all three techniques could identify the patients with <10% of normal proliferation. They additionally highlighted that thymidine incorporation is seemingly more advantageous than the other two techniques, since the quantitative value of [^3^H] thymidine is more easily recognized and its execution does not rely on skilled operators [13]. 

At the same time, Ki-67^+^ appears to be a more sensitive marker, since its expression identified almost two times the frequency of proliferating T cells than was detected by the BrdU assay [43], probably since the incubation of BrdU-stained cells can only be limited to a shorter incubation time of 24 h. Soares and colleagues also observed a strong and significant correlation between Ki-67^+^ CD4^+^ T cell expression and BrdU incorporation (*r* = 0.8 to 0.93, *p* < 0.0001), as well as with the dye dilution of Oregon Green, a CFSE derivative (*r* = 0.95 to 0.99, *p* < 0.0001) [43]. 

#### 2.2.4. Colorimetric Assay(s)

The proliferation of cells in culture can also be determined by measuring the formation of a reduced nicotinamide adenine dinucleotide (NADH) and reduced nicotinamide adenine dinucleotide phosphate (NADPH) content. With the use of these pyridine nucleotides or biochemical markers, a direct but not absolute measurement that acts as an indicator for metabolic activity is possible [95]. By using NADH and NADPH as electron sources, different tetrazolium salts such as water-insoluble formazan, 3-(4,5-dimethyl thiazol-2-yl)-2,5-indiphenyl tetrazolium bromide (MTT), water-soluble formazan, or water-soluble tetrazolium salt (WST) can be enzymatically reduced, resulting in a color change that can be measured by basic spectroscopic methods [95].

##### 3-(4,5-Dimethyl thiazol-2-yl)-2,5-indiphenyl Tetrazolium Bromide (MTT) Colorimetric Assay

The use of 3-(4,5-dimethyl thiazol-2-yl)-2,5-indiphenyl tetrazolium bromide (MTT), a tetrazolium salt, in an MTT assay was first introduced by Mosmann in 1983 as a quantitative rapid colorimetric assay to track cellular survival and growth [96]. Similar to CFSE, the MTT reagent can pass through the cell membrane and the mitochondrial inner membrane of cells due to its lipophilic structure and positive charge [97]. Mitochondrial dehydrogenase enzymes in metabolically active cells [98] reduce MTT by disrupting the core tetrazole ring, forming formazan—a violet-blue water-insoluble molecule—in the process [99]. With the involvement of glycolysis-induced NAD(P)H generation, the MTT assay, as such, directly correlates the number of viable cells present by determining the amount of oxidation produced. The intracellular production of formazan hence provides a foundation for colorimetric-based measurements in MTT assays [100]. 

The lack of radioisotope use and the absence of washing steps allowed the MTT assay to be recognized as a rapid, yet precise, method for measuring cytotoxicity by detecting the presence of living cells, proliferation, and/or activation [96]. As a result, the MTT assay has since been applied across several functional tests in both humans and animals, including cytokine and cytotoxicity assays as well as mitogen-induced lymphocyte blastogenesis [41,96]. By comparing the MTT assay with the [^3^H] thymidine LPT, a very good correlation [41] was reported following the mitogenic stimulation of PBMCs by PHA. 

However, inconsistencies in confounding variables that have been identified across several studies, such as the seeding cell number, the concentration of MTT reagent added, the length of incubation, the culture media type, and the wavelength at which the optical density (OD) is measured by an ELISA microplate reader, contribute towards the difficulty of comparing results between different studies [100]. The MTT assay is also limited as opposed to other assays, as it assesses proliferation by comparing the estimated rather than absolute cell numbers at various time points and/or conditions. A complete understanding of the underlying mechanisms of the MTT assay is also incomplete due to the many uncertainties revolving around certain aspects of this assay, such as the type of enzymes, molecules, and organelles involved in MTT reduction, the cytotoxic effect of MTT, and most importantly, how the assay measurements represent or act as an indicator for cell viability and metabolic activity on top of lymphoproliferation [97].

### 2.3. Future Perspectives

Dye-based flow cytometric proliferation assays still dominate among the array of LPT alternatives introduced into the field thus far. In contrast to other approaches that only score proliferation, dye-based proliferation assays offer the added possibility of retrieving information on top of the overall proliferative response, such as the frequency of antigen-specific lymphocyte precursors and the phenotypical characterization of cells at different stages of proliferation. In the near future, additional attention to fluorescence-activated cell sorting (FACS) and flow cytometry analytical techniques could therefore improve our current understanding of the extent to which T and/or B cell proliferation contributes towards overall lymphoproliferation. 

## 3. Concluding Remarks

Over the last decades, various LPT alternatives with fluorescent labelling approaches and/or immunophenotyping, as well as colorimetric assays, have been proposed to track lymphocyte proliferation. In some cases, alternatives such as MELISA^®^, FASCIA, and immuno-BeLPT have repurposed classic techniques that rely on the use of radioactive isotopes or have merged two or more known techniques which, in turn, allowed for improvements in the tests’ performances. 

At this time, the use of fluorescent dye CFSE in a CFSE assay particularly stands out as the most multifunctional and versatile LPT alternative, in light of its ability to homogenously label both proliferating and parent populations and quantify lymphocyte proliferation based on customizable parameters, and its cost-effectiveness in comparison to the [^3^H] thymidine LPT. In fact, in a handful of comparative studies, the correlation of techniques was compared in conjunction with the CFSE assay rather than with the [^3^H] thymidine LPT, which, to a certain extent, could be indicative of a progressive shift towards a more accessible and reliable alternative, unlike the classic LPT. 

Concurrently, we cannot disregard the fact that the preferential selection of any of the included LPT alternatives would vary based on the suitability for the experiments in mind. Factors such as the necessity to track PBMC survival over time per lymphocyte subset, favoring certain spectral properties unlike CFSE or VPD-450, the homogenous labelling of lymphocytes as a prerequisite, and the priority for short-term or long-term analyses are some of the reasons that can contribute towards the reasoning and the subsequent disparity observed in selecting an ideal LPT technique. Regardless, we postulate that though the CFSE assay may not always be regarded as the best alternative to the [^3^H] thymidine LPT, it can and should be considered as the better LPT alterative.

## Figures and Tables

**Table 1:** Comparative studies across various techniques that measure lymphocyte proliferation.

Methods Compared	Types of Analysis	Stimulants	Groups	Observations	References
LPT, CFSE assay, and Ki-67^+^ expression	Correlation performed using SPSS	ConA, PHA, PWM, BCG, and *C. albicans*	9–19 controls	Non-significant correlation between the 3 methods Correlation ranged from *r* = 0.2–0.9, 0.2–0.8, 0.1–0.8, 0.2–0.7, and 0.2–0.9 for ConA, PHA, PWM, BCG, and *C. albicans*, respectively LPT is most sensitive	[13]
LPT and VPD-450	Wilcoxon signed rank test, Spearman’s rank correlation (non-parametric), and mean of control + x times SD	Hemagglutinin peptide pools of peptides	4 controls (vaccinated)	Strong correlation between both assays based on percentage of positive wells (*r* = 0.815, *p* = 0.02) and SI > 2 (*r* = 0.83, *p* = 0.01) Mean of control + x times SD, would be the optimal cut-off for detecting a positive response without misinterpretation of the results	[1]
LPT and MELISA^®^	Spearman’s rho correlation	Beryllium	4 sarcoidosis patients	Strong/perfect correlation of 1.00 (*p* < 0.01) between beryllium SI of MELISA^®^ and beryllium LPT	[42]
LPT and VPD-450	Spearman’s rank correlation	Allogenic antigens, *C*. *albicans*, TT, and *V*. *zoster* virus	3 controls	Good correlation between VPD-450 precursor frequency (%) and [^3^H] thymidine incorporation with allogenic (*r* = 0.91, *p* < 0.0001) and pathogen-derived antigens	[7]
LPT and FASCIA	Correlation was performed, but statistical test was unspecified	PHA, PWM, and ConA	12–19 controls	Good correlation: *r* = 0.78 was observed with LPT-cpm and number of lymphoblasts with FASCIA for all stimuli	[40]
LPT and CFSE assay	Linear regression model was applied and Pearson’s correlation was calculated	PT antigen and FHA	29 controls	Significant and strong correlation between % divided of CFSE^dim^ and LPT-cpm with PT antigen (*r* = 0.691, *p* = 0.001), but not with FHA (*r* = 0.198, *p* = 0.430)	[30]
LPT and CFSE assay	Spearman’s rank correlation	Spontaneous proliferation	45 HTLV-1-infected patients and 14 controls	Positive correlation was found between the division index of cells and [^3^H] thymidine incorporation (*r* = 0.84; *p* = 0.001), as well as between the percentage of divided cells and [^3^H] thymidine incorporation (*r* = 0.78; *p* = 0.004)	[31]
BrdU, Ki-67^+^ expression, and Oregon Green—a CFSE derivative	Spearman’s rank correlation	PPD and TB10.4 protein	15 controls	Strong and significant correlation between Ki-67^+^ CD4^+^ T cell expression and BrdU incorporation with *r* = 0.8–0.9.	[43]
LPT and CFSE assay	Spearman’s rank correlation	PHA	128 patients with various immunological disorders and 40 controls	Highest correlation of *r* = 0.807 with LPT-cpm was observed for analysis of % divided T cells (CD3^+^, but non-significant) and *r* = 0.776 (and significant), was observed with % divided total lymphocyte population	[32]
LPT and CFSE assay	McNemar and Fisher tests, kappa statistic, Bland–Altman, and mountain plots	Beryllium	38 Be-hyper-sensitive (7/38 with CBD), 22 Be-sensitive, and ~12 controls	Based on kappa statistic; good (0.627–0.805) and significant agreement between methods Significant correlation with Bland–Altman plots (although values of significance not reported) Mountain plots indicated that the methods were unbiased with respect to each other	[33]
LPT and ELISPOT	Linear relation; analytic performance of both assays was evaluated using AUC of ROC curves	HER-2/neu, TT, and CMV	27 breast cancer patients	R^2^ of 0.428 for CD4^+^ T cell proliferation and R^2^ of 0.531 for interferon-gamma (IFN-γ) secretion Sensitivity of LPT and ELISPOT assessed across the 3 antigens (sensitivity, AUC, and *p*-values reported)	[44]
Red fluorescent dye SNARF-1, LPT, and CFSE assay	Pearson correlation (parametric)	PHA or OKT3 monoclonal antibody	Controls	SNARF-1 labelling yielded comparable results for T cell proliferation when compared to CFSE assay (*r* = 0.40 for PHA and *r* = 0.99 for OKT3. When compared with LPT, correlation with SNARF-1: *r* = 0.62 for PHA and *r* = 0.97 for OKT3; with CFSE: *r* = 0.84 for PHA and *r* = 1.0 for OKT3	[34]
LPT and CFSE assay	Spearman’s rank correlation	Autologous Tregs	11 multiple sclerosis patients and 5 controls	Significant and strong correlation between suppression levels of Tregs obtained by CFSE assay and [^3^H] thymidine incorporation (*r* = 0.91–0.92, *p* < 0.01)	[35]
LPT and CFSE assay	No direction comparison was made	antigen KLH—type of vaccine taken up by dendritic cells	6 renal cell carcinoma patients	Consistent results observed with both tests	[36]
LPT and CFSE assay	Wilcoxon signed rank test and fold difference in sensitivity to antigen	Tt, GAD	10 controls	CFSE yielded stronger responses than LPT (*p* = 0.01) Capacity of each assay to detect proliferation was compared across concentrations	[37]
LPT and BrdU assay	Pearson correlation	Anti-CD3/CD28	26 HIV-1-infected patients and 18 controls	Strong correlation observed for BrdU assay with LPT at *r* = 0.82, 0.83, and 0.96 with overall (HIV^+^ and HIV^-^ individuals), HIV^-^ individuals, and HIV^+^ individuals, respectively	[45]
LPT, CFSE assay, and CD69^+^ expression	Correlation was performed, but statistical test unspecified	PHA	2 patients with T cell deficiency and 11 controls	CFSE-derived values correlated well (good correlation) with the peak [^3^H] thymidine uptake/cpm	[38]
LPT, CFSE assay, and CD69^+^ expression	Linear and polynomial fitted correlation	*C. albicans*	6 patients with previous episodes of vaginal candidiasis (exposed), 3 patients with CRVVC, and 7 controls with undocumented Candida infection (unexposed)	Strong/close correlation observed with *r*^2^ = 0.93, 0.99, 0.95, and 0.82 when weighted division index was compared with % blasts, weighted % divided, division index, and CD69 increment, respectively	[39]
LPT, BrdU, MTT, and NAG assay	Correlation assessed by multilinear regression analysis	PHA	Controls	Strong correlation, *r* = 0.89 between OD of BrdU incorporation and LPT-cpm, while MTT test revealed higher SI values	[46]

Legend (in order of appearance): LPT: lymphocyte proliferation test, CFSE: carboxyfluorescein succinimidyl ester, ConA: concavalin A, PHA: phytohemagglutinin, PWM: pokeweed mitogen, BCG: Bacillus Calmette–Guérin, *C*. *albicans*: *Candida albicans*, TT: tetanus toxoid, VPD-450: violet proliferation dye 450, SD: standard deviation, MELISA^®^: memory lymphocyte immunostimulation assay, *V. zoster* virus: *Varicella zoster* virus, FASCIA: flow-cytometric assay for specific cell-mediated immune response in activated whole blood, cpm: counts per minute, PT: pertussis vaccinal antigens, FHA: filamentous hemagglutinin, HTLV-1: human T lymphotropic virus type 1, BrdU: bromodeoxyuridine/5-bromo-2′-deoxyuridine, PPD: purified protein derivative, Be: beryllium, CBD: chronic beryllium disease, ELISPOT: enzyme-linked immunosorbent spot, AUC: area under curve, ROC: receiver operating characteristic, HER-2/neu: human epidermal growth factor receptor 2, CMV: cytomegalovirus, IFN-γ: interferon-gamma, SNARF-1: Seminaphtharhodafluor-1, KLH: keyhole limpet hemocyanin, GAD: glutamic acid decarboxylase, HIV-1: human immunodeficiency virus 1, cpm: counts per minute, CRVVC: chronic recurrent vulvovaginal candidiasis, MTT: 3-(4,5-dimethyl thiazol-2-yl)-2,5-indiphenyl tetrazolium bromide, NAG: *p*-nitro-phenol-*N*-acetyl-~-d-glucosaminide, OD: optical density.

**Table 2:** Reported drawbacks and benefits of the [^3^H] thymidine LPT and CFSE assay.

[^3^H] Thymidine LPT	CFSE Assay
Radioactive reagents must be handled and disposed of with caution [60]	CFSE fluorescence precisely halves after each cell division in a highly predictable manner and is thus highly amenable to mathematical modelling [61]
Thymidine is measured by the scintillation counter per well of cells rather than per individual cell, so the assay reveals nothing about an individual cell’s division history [60]	CFSE is able to discriminate between the proliferation of regulatory T (Treg) and target cells in an experimental setting where Treg cell suppression capacity is measured by its potential to inhibit target cell proliferation [35]
No additional assays can be performed with or after [^3^H] thymidine incorporation, since it is an endpoint assay [60]	Several studies have shown that CFSE labelling can be toxic for cells [32,62], not just in terms of cell death, but also in terms of cell function—the ability of the cells to proliferate can be seriously compromised [61]
The inability to identify cells that have undergone numerous rounds of division [60] restricts clarity on the type of proliferating cells [63]	The use of total PBMCs could be theoretically more informative, since it will allow studying the response of different lymphocyte sub-populations [7]

## Data Availability

Not applicable.

## Abbreviations

LPT: lymphocyte proliferation test, PBMCs: peripheral blood mononuclear cells, cpm: counts per minute, SI: stimulation index, BL: borderline, HSCT: hematopoietic stem cell transplantation, TREC: T cell receptor excision circle, BeS: beryllium sensitivity, CBD: chronic beryllium disease, Be: beryllium, BeO: beryllium oxide, BeLPT: beryllium lymphocyte proliferation test, BAL: bronchoalveolar lavage, NiLPT: nickel lymphocyte proliferation test, MELISA^®^: memory lymphocyte immunostimulation assay, BrdU: bromodeoxyuridine/5-bromo-2′-deoxyuridine, EdU: ethynyldeoxyuridine/5-ethynyl-2′-deoxyuridine, CFSE: carboxyfluorescein succinimidyl ester, FASCIA: flow-cytometric assay for specific cell-mediated immune response in activated whole blood, VPD-450: violet proliferation dye 450, CD: clusters of differentiation, HLA-DR: human leukocyte antigen—DR isotype, MTT: 3-(4,5-dimethyl thiazol-2-yl)-2,5-indiphenyl tetrazolium bromide, mAbs: monoclonal antibodies, DAPI: 4′6′-diimidazolin-2-phenylindole, PI: propidium iodide, MFI: median fluorescence intensity, PHA: phytohemagglutinin, PT: pertussis vaccinal antigens, TT: tetanus toxoid, *C*. *albicans*: *Candida albicans*, CMV: cytomegalovirus, FITC: fluorescein isothiocyanate, GFP: green fluorescence protein, *V. zoster* virus: *Varicella zoster* virus, NADH: nicotinamide adenine dinucleotide, NADPH: nicotinamide adenine dinucleotide phosphate, FACS: fluorescence-activated cell sorting.
